# Sequential Fragmentation of Pleistocene Forests in an East Africa Biodiversity Hotspot: Chameleons as a Model to Track Forest History

**DOI:** 10.1371/journal.pone.0026606

**Published:** 2011-10-28

**Authors:** G. John Measey, Krystal A. Tolley

**Affiliations:** 1 Applied Biodiversity Research Division, South African National Biodiversity Institute, Cape Town, South Africa; 2 Department of Biodiversity and Conservation Biology, University of the Western Cape, Bellville, South Africa; 3 Department of Botany and Zoology, University of Stellenbosch, Stellenbosch, South Africa; Centre National de la Recherche Scientifique, France

## Abstract

**Background:**

The Eastern Arc Mountains (EAM) is an example of naturally fragmented tropical forests, which contain one of the highest known concentrations of endemic plants and vertebrates. Numerous paleo-climatic studies have not provided direct evidence for ancient presence of Pleistocene forests, particularly in the regions in which savannah presently occurs. Knowledge of the last period when forests connected EAM would provide a sound basis for hypothesis testing of vicariance and dispersal models of speciation. Dated phylogenies have revealed complex patterns throughout EAM, so we investigated divergence times of forest fauna on four montane isolates in close proximity to determine whether forest break-up was most likely to have been simultaneous or sequential, using population genetics of a forest restricted arboreal chameleon, *Kinyongia boehmei*.

**Methodology/Principal Findings:**

We used mitochondrial and nuclear genetic sequence data and mutation rates from a fossil-calibrated phylogeny to estimate divergence times between montane isolates using a coalescent approach. We found that chameleons on all mountains are most likely to have diverged sequentially within the Pleistocene from 0.93–0.59 Ma (95% HPD 0.22–1.84 Ma). In addition, post-hoc tests on chameleons on the largest montane isolate suggest a population expansion ∼182 Ka.

**Conclusions/Significance:**

Sequential divergence is most likely to have occurred after the last of three wet periods within the arid Plio-Pleistocene era, but was not correlated with inter-montane distance. We speculate that forest connection persisted due to riparian corridors regardless of proximity, highlighting their importance in the region's historic dispersal events. The population expansion coincides with nearby volcanic activity, which may also explain the relative paucity of the Taita's endemic fauna. Our study shows that forest chameleons are an apposite group to track forest fragmentation, with the inference that forest extended between some EAM during the Pleistocene 1.1–0.9 Ma.

## Introduction

We are only beginning to unravel the processes that have produced high species richness and endemism in particular areas. The formation of hypotheses to explain elevated species richness originated with studies of the once fragmented, now continuous tropical forests in Central and South America [1]. Some extant tropical forests are naturally fragmented habitats and their climatically facilitated break-up has been widely seen as the source for increased forest biodiversity through vicariant speciation [1], [2], [3], , as has climatically induced fragmentation in many temperate areas [5], [6], [7]. Indeed, there is growing evidence that natural fragmentation and merging of tropical forests may be a cause of elevated biodiversity [4], [8], [9], [10]. Vicariant speciation across spatially fragmented habitats however, is likely to be directly related to each species' ability to disperse between habitat fragments. In the absence of direct paleo-data detailing the presence of ancient forests, studies attempting to investigate forest fragmentation must be based on model species which require continuous forest for their dispersal.

The Eastern Arc Mountains and coastal forests of Tanzania and Kenya (hereafter EAM) are an excellent example of naturally fragmented tropical forests which are of particular interest as they contain one of the highest known concentrations of endemic plants and vertebrates on earth [11], [12]. The EAM qualify as a biodiversity hotspot as they exhibit excessive endemism with highly nested distribution patterns [8], in addition they have been recognised as being in need of concerted conservation efforts [12], [13]. Large montane blocks have been the traditional focus of surveys and conservation priorities within the EAM [14], but increasing surveys into smaller isolates continue to discover restricted endemic species, even when these are close to well surveyed areas [15], [16], [17], [18] demonstrating the importance of even the smallest forested isolates. East Africa has also been the subject of numerous paleo-climatic studies, as past climate change has been linked with the advancement of early hominid lineages, revealing multiple wet-dry cycles as a result of Milankovitch climate forcing [19]. However, the range of dates for which cycles have been recorded is vast; as recent as the last glacial maximum to periods which pre-date the formation of the EAM.

Trauth et al [20] studied diatom assemblages in sediment cores of lakes in the East African Rift system demarcating three wet periods during continuing post-Miocene aridification in East Africa: 2.7–2.5, 1.9–1.7 and 1.1–0.9 Ma. Moreover, wet-dry cycles continued throughout the Pleistocene recorded in glaciation events on Kilimanjaro and Mt. Kenya which coincide with recent glaciation events at northern latitudes up to and including the last glacial maximum (21±2 Ka) [21]. Climate fluctuations have been inferred from studies on volcanoes throughout the region up to 140 Ka[22], [23], [24], [25], together with commensurate wet-dry cycles in low-lying areas [26].

Pollen cores have been particularly useful in determining the reorganisation of vegetation during climatic shifts which includes altitudinal variation and expansion of forest [24]. In marked contrast, core sediments taken from montane swamps within the Eastern Arc Mountains suggest little climate fluctuation in the last 50 Ka [27], [28], [29] corroborating earlier ideas that these highland areas have remained humid, stable environments [3]. However, it is still unknown what effects recent cycling through wet and dry periods has had on the vegetation of the surrounding lowlands. With continuous presence of montane forest, wet periods may have produced lowland forests allowing the movement of forest dependant taxa between composite blocks of the EAM. Indeed, this hypothesis has been invoked in explaining how the complex vicariant nature of high species diversity in the area is possible ([30] and references therein). Because of the overall complexity of the EAM system, obtaining a confident estimate for the last period when forests were connected between mountain blocks would provide a sound basis for hypothesis testing on vicariance and dispersal models.

The use of phylogenetic methods, and in particular coalescent modelling, has allowed researchers to estimate significant periods when clades of animals and plants diverged. These investigations allow a different perspective on the numerous periods of climatic fluctuations in East Africa, highlighting those which have been of sufficient magnitude to allow dispersal (or vicariant) events. Although there is a consistent signal which demonstrates that Pleistocene climatic oscillations were important in structuring many birds [31], mammals [32] and amphibians [33], no common patterns have yet emerged [8], [34], [35]. Indeed, the complex nature of connectivity and dispersal scenarios even demonstrate differences in divergence times of co-generic taxa [36]. It is clear that obtaining an estimate of the last period of significant forested connection requires consensus from many taxa, but with special attention to taxa which are forest dependant without cryptic dispersal through arid areas.

Chameleons are traditionally thought of as a taxon with poor dispersal abilities, which made reports of their long distance dispersal to the Seychelles from Africa particularly interesting [37]. Gene-flow in South African dwarf chameleons (*Bradypodion* spp.) inhabiting the western Cape Floristic Region (another biodiversity hotspot), is thought to be historically low [38]. In addition, chameleons are often restricted to particular vegetation types [39], [40], and in East Africa, the speciose genus *Kinyongia* is associated exclusively with forested habitats [41], [42]. Recently, Tolley et al. [43] demonstrated that most of the lineages are ancient, having radiated in the early Miocene or Oligocene, and that this matches the pattern of pan-African forest fragmentation on a course scale [2].

**Figure 1:**
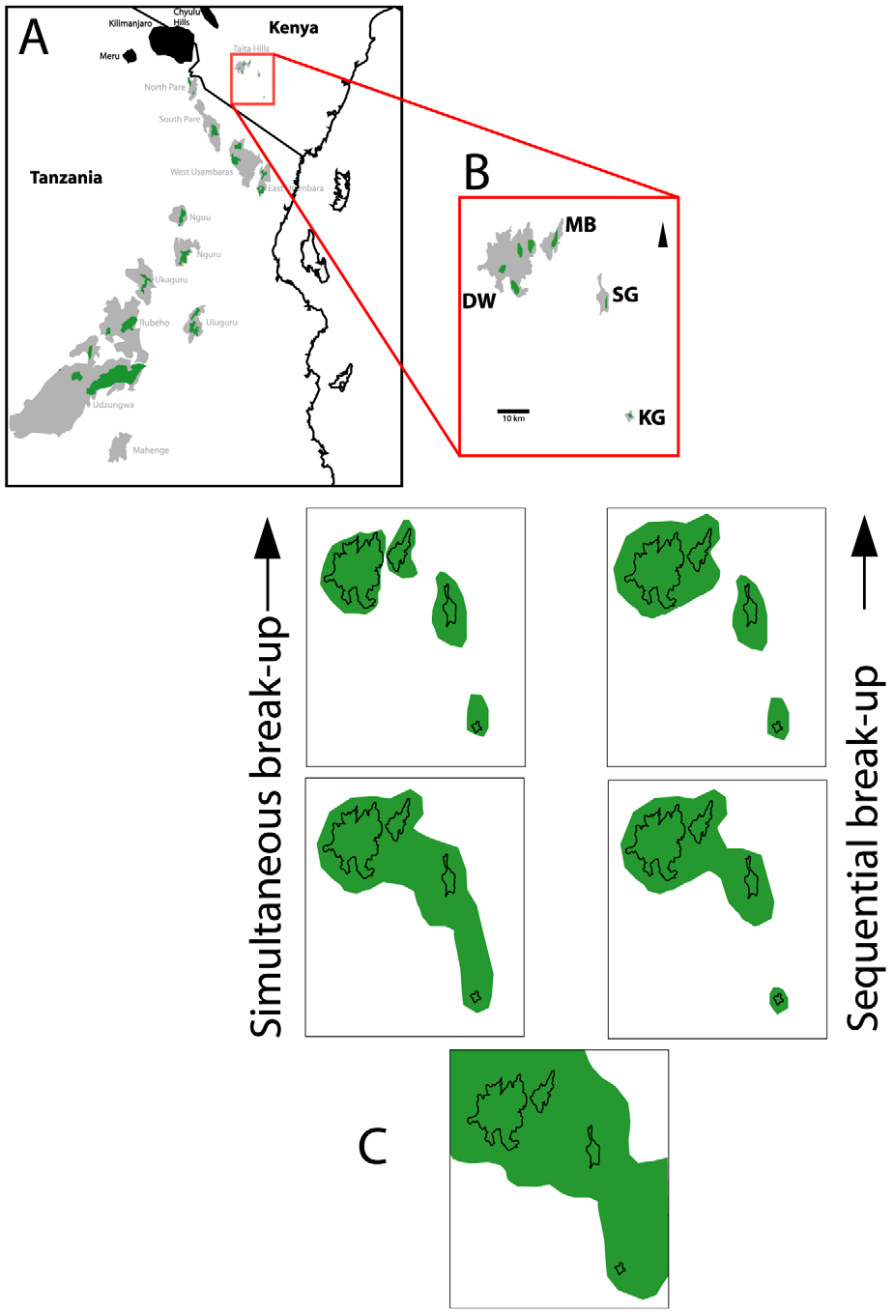
Two potential vicariance scenarios for break-up of forests. A and B show pathways of different break-up scenarios in the northernmost component of the C) Eastern Arc Mountains (shown in grey with volcanic mountains in black): Dawida (DW), Mbololo (MB), Sagalla (SG) and Kasigau (KG) make up the Taita Hills (D). A) shows forest (in green) shrinking around the mountains with eventual fragmentation in lowland areas occurring simultaneously.(B) shows a scenario whereby forest (in green) on the most distant mountain (Kasigau – see D) is the first to separate and the other mountains follow successively.

To investigate forest biogeography on a fine scale, we focus on intraspecific gene-flow of the two-horned chameleon, *Kinyongia boehmei* endemic to the Taita Hills complex of the EAM. The four forest covered montane isolates that make up the Taita Hills are all within an area of 33 km^2^, separated by tracts of dry savannah from 4 to 50 km, and can be regarded as a model for studying dispersal and vicariance on a small geographic scale within the EAM. We hypothesised that the arid savannah between montane isolates would allow us to date the last period during which gene-flow for chameleons was possible via tracts of forested lowlands. Two vicariance scenarios were considered, whereby montane forests either became isolated simultaneously during aridification or sequentially in relation to their respective distances (Fig. 1).

## Results

**Figure 2:**
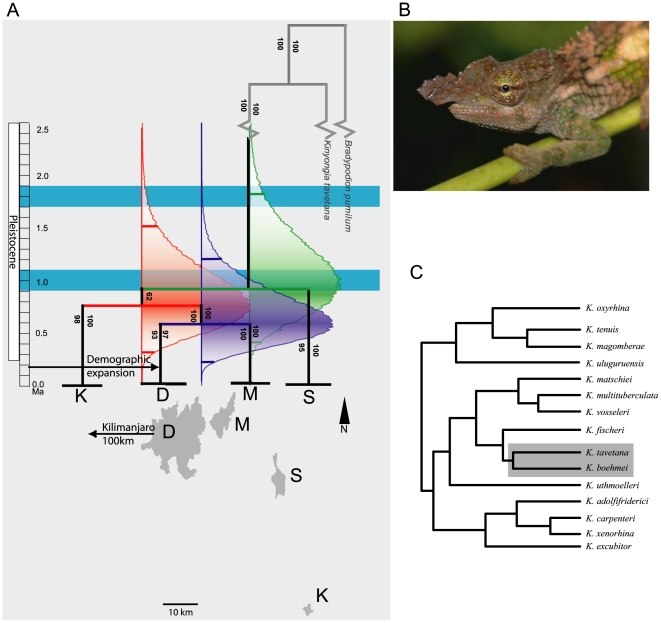
The phylogenetic position of *Kinyongia boehmei* and its temporal divergence in the Taita Hills, Kenya. A) Topology for the prior used in the coalescent analysis for dating {Hey, 2010 #1301} of *K. boehmei* from the Taita Hills. Confidence in this topology prior was estimated by Bayesian posterior probabilities (to the left of each branch), and maximum likelihood boostrap (to the right). Divergence times from IMa2 (scale on left in Mya) are given as 95% highest posterior density (HPD) and their high points (horizontal red, green and blue bars with respective HPD distributions). Horizontal blue bars represent two wet periods in the Pleistocene proposed by Trauth et al [24]. Arrows show time timing of a demographic expansion in Dawida (above), and the relative position of Kilimanjaro to the Taita complex (below: D Dawida; M Mbololo; S Sagalla; K Kasigau). B) A male *Kinyongia boehmei* in life with C) topology for the genus *Kinyongia* showing the relative position of to the outgroup (*K. tavetana*) and other members of the genus from Tolley et al [47].

**Figure 3:**
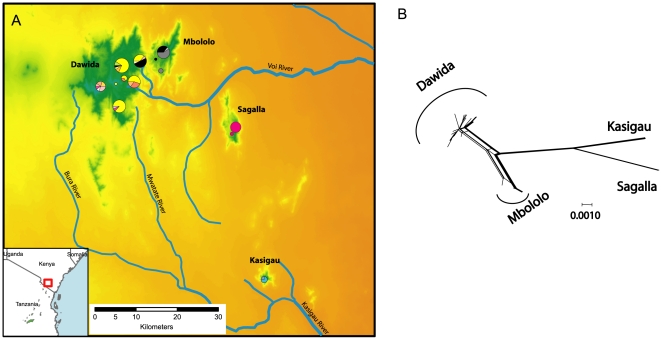
Sampling localities, haplotype frequencies and haplotype network for *Kinyongia boehmei* from the Taita Hills, Kenya. A) The distribution of sampling localities within the Taita Hills, Kenya (inset). Topography is shown from green (highest) to orange (lowest) with major rivers labelled. Circles are proportional to number of individuals sampled and segments within the circles represent haplotypes. Note the presence of Mbololo haplotypes (black) in the northern areas of Dawida. B) ND2 network visualised using the NeighborNet algorithm with secondary contact omitted. Each line represents the connection between two haplotypes with the thicker lines representing many connections.

Chameleons from each of the four montane isolates could be grouped into a well-supported clade (Fig. 2A) with Dawida and Mbololo supported as sister clades. The Bayesian and ML consensus topologies, which were used as a prior for the coalescent analysis, were identical (Fig. 2A). The SAMOVA analysis showed an unambiguous interpretation for four populations with the maximum value of F_CT_ (0.89), corresponding to each of the four montane isolates. Haplotype (*h*) and nucleotide (π) diversity were greatest for the population on Dawida followed by Mbololo, Kasigau, and Sagalla (Table 1). No haplotypes were shared between mountains, except for the presence of a common haplotype (35% of the samples) on Mbololo which was shared with 9 individuals (8% of samples) from Dawida (Fig. 3). The shared “Mbololo haplotype” is particularly divergent (average of 10 mutational steps) from the majority (92%) of the Dawida chameleons sampled. We interpreted this pattern as an occurrence of recent “secondary contact” whereby individuals from Mbololo have been transported to Dawida, rather than shared ancestral polymorphism or historical gene flow. The population level analysis (SAMOVA) was therefore run again without these “secondary contact” individuals, and the result similarly suggested the same four populations (F_CT_ = 0.79). Although the sharing of these haplotypes due to ancestral polymorphism or a natural contact zone cannot be discounted, we considered those scenarios unlikely for reasons discussed below.

**Table 1:** Haplotype and nucleotide diversity for populations of *Kinyongia boehmei* from the Taita Hills.

	Dawida (no sc)	Dawida (sc)	Mbololo	Sagalla	Kasigau
n	99	108	17	15	4
ND2 Haplotypes	17	18	3	1	2
Haplotype	0.6887	0.7324	0.5588	0.0000	0.5000
Diversity (*h*)	±0.0504	±0.0438	±0.0831	±0.000	±0.2652
Nucleotide	0.0016	0.0037	0.0008	0.0000	0.0006
Diversity (π)	±0.0012	±0.0023	±0.0008	±0.0000	±0.0008

Note that two results are provided for Dawida: with (sc) and without (no sc) presumed secondary contact (see text for details).

Uncorrected p-distances between mountains ranged between 0.5–2.0% for 16S, 1.4–3.5% for ND2, 0–0.3% for phosducin, and 0.05–0.5% for RAG1. Although these values are slightly high for population level divergences, they are lower than typically expected between chameleon species [41], [44], [45], [46], [47]. The values are not unexpected for historically isolated populations within a single species.

Only the population from Dawida (with secondary contact individuals removed) was found to be out of mutation-drift equilibrium with a significant negative value for *F_S_*, suggesting a demographic shift in the population, such as population expansion (Tajima's D = −1.8, p<0.05; Fu's *F_S_* = −11.2, p<0.001). The value obtained for τ = 1.61 (95% CI: 0.0–4.33), provided an estimate for *t* of ca. 91 000 generations or ca. 182 000 years since the inferred demographic expansion on Dawida. Isolation by distance was not found between these four mountains (*p* = 0.37) suggesting that proximal forests were not connected more recently than more distant forests, and that some factor other than linear geographic distance maintained forest connections.

### Divergence times

Mutation rates used to estimate divergence times were obtained for three markers (16S, 2.1×10^−9^; ND2, 5.7×10^−9^; RAG 4×10^−10^), with phosducin excluded here as it was not included by Tolley et al [43] precluding a mutation rate estimate. The estimates of divergence time reveal three different periods during which divergence occurred, although 95% HPDs were overlapping. The highest point for the HPD for each of the divergence events was used as an indication of the most likely divergence scenario. This indicated that divergence between Dawida and Mbololo was most recent, at ca. 0.6 Ma (95% HPD = 0.22–1.21 Ma), preceded by divergence between Kasigau and Dawida+Mbololo complex (0.76 Ma, HPD = 0.32–1.53 Ma), with the oldest divergence time between Sagalla and all other isolates (0.93 Ma, HPD = 0.41–1.84 Ma; Fig. 2A).

## Discussion

Our results from forest chameleons in the Taita Hills demonstrate isolated populations on top of each montane isolate, within a single species. Divergence between populations is not contemporaneous but neither does it follow a simple isolation by distance model. Divergence time estimates suggest that chameleons on Sagalla have been isolated from the other mountains longer, despite proximity to Mbololo and Dawida, whereas connections were maintained between the more distant Kasigau chameleons with those on Mbololo and Dawida. Fragmentation of the closest and most recently diverged montane isolates (Dawida and Mbololo) predates the most recent wet-dry cycles since the last glacial maximum [21] and the oldest divergences correspond to the last significant wet episode during post-Miocene aridification in East Africa [48].

Multi-gene phylogenetic and population level approaches support four populations of *Kinyongia boehmei*. The estimated date at which populations of *K. boehmei* on the closest isolates (Mbololo and Dawida, 4 km apart) diverged in the mid-Pleistocene (0.59 Ma; HPD 0.22–1.21 Ma). As these chameleons are confined to forested and wooded areas, and the genus has never radiated into savannah habitat [43], we interpret this divergence time as the last period that chameleons moved between montane isolates signifying the final fragmentation of forest patches. Despite the small distance that separates these mountains, chameleon populations are clearly divergent, suggesting that narrow, but deep valleys were not forested during the most recent wet-dry cycles, *circa* 21 Ka [21]. Instead, divergence times are on longer time scales corresponding to aridification over the entire area following the last wet period 0.9–1.1 Ma [48]. In addition, our data add credence to the hypothesis that the EAM have acted as ancient forested refuges [8], despite paleontological evidence for forest covering only the last 50 Ka [27], [28], [29]. Indeed, Mount Kasigau with a forest area of only 216 ha [49] has maintained a distinct lineage of *Kinyongia boehmei* for around 0.76 Ma (HPD = 0.32–1.53 Ma), and underlines the importance of the even smallest EAM inselbergs in producing the highly nested distribution patterns characteristic of this biodiversity hotspot of excessive endemism [8].

Forest connections between the other montane isolates of the Taita complex ended without reforming in the mid-Pleistocene according to our coalescent analysis. The highest probability for each divergence time follows the end of the most recent wet period embedded within an overall pattern of post-Miocene aridification in East Africa: 0.9–1.1 Ma [48]. However, there remains a small probability (<95%) that the last connection for Sagalla and the other montane isolates dates back to the older wet period: 1.7–1.9 Ma [48]. We speculate that this older wet period would also likely have joined all forests that were previously fragmented on each isolate, so that the younger represents a wet period when forests first re-establish between Sagalla, Kasigau, Dawida and Mbololo around 1.1 Ma and then fragmented again (from 0.9 Ma). We infer that gene-flow of chameleons (and other forest restricted taxa) would have occurred during each of these wet periods of similar magnitude throughout the Pleistocene [20], [48].

The strong population level divergences observed suggest that the arid savannah prevents contemporary gene-flow. These results suggest that the two closest mountains (Dawida and Mbololo) are closely related, but that these are sister to the most distant isolate of Kasigau (50 km south: Fig. 3A) rather than those from the much closer Sagalla (22 km east). Indeed, neighbouring EAM (East and West Usambaras, South Pare) are less than 50km from Dawida/Mbololo (Fig. 1A), yet different species of *Kinyongia* are found on these mountains [43]. The pattern is similar to that of caecilians (Amphibia: Gymnophiona) which occur on all four isolates of the Taita Hills [50], [51] but the lineage from Sagalla is a sister species to that on Dawida, Mbololo and Kasigau [18], [33].

The pattern shown by both chameleons and caecilians suggests that a corridor between Dawida/Mbololo and Kasigau remained intact longer than any connection between Sagalla and the other component isolates. This putative corridor corresponds well with river beds that run between Dawida and the southeastern aspect of Kasigau where the monsoonal orographic effect is greatest (see Fig. 3A). At present, these rivers flow only periodically (GJM pers. obs.), but in wetter periods it is likely these rivers were well established, and supported riverine forest allowing a continuous connection between these distant montane isolates. In contrast, the Voi River, runs continuously from Dawida but passes to the dry northwestern aspect (i.e. within the rain shadow) of Sagalla, so that even in wetter periods, there may have been no connection between riverine and montane forest. Riverine corridors have previously been used to explain the distribution and dispersal of amphibians in East Africa [33], [35], [52], and it is possible that other complex dispersal scenarios in this region [36] might be best explained with riverine corridors.

The idea that forest occurred between components of the Eastern Arc Mountains during the mid-Pleistocene, some 0.9–1.1 Ma corresponds with expansions in other faunal groups [53], [54]. However, this period also saw major normal faulting producing the present-day rift escarpments and changing previous watershed directions [55]. This important tectonic activity was accompanied by large and sustained volcanic activity in close proximity to the Taita Hills. Thus, both climatic shifts and changes in watersheds are tightly linked to this period.

The lack of mutation-drift equilibrium and significant negative *F_S_* value in the Dawida population suggests there was a demographic expansion in this area. We propose that, as the chameleon population on Dawida maintains marked genetic independence from the other montane isolates, a population expansion occurred on Dawida from a small population that may have survived a catastrophic event. Volcanic eruptions are good candidates for such events as the area is within the North Tanzanian Divergence [56] with nearby volcanic centres Chyulu and Kilimanjaro. Our data shows that the chameleon population on Dawida expanded some 182 Ka, while the last major eruption of Kilimanjaro can be dated to the same period: ca. 200–150 ka [57]. The last eruption at Kilimanjaro is likely to have been the single biggest event in the area. Such volcanic activity may have had large local impacts on the Taita Hills, especially Dawida which is the closest of the four inselbergs to Kilimanjaro (100 km west, see Fig. 1A). This explanation may also account for the low herpetofaunal diversity in the Taita Hills compared to other Eastern Arc Mountains [50].

It is possible that chameleons on Sagalla were also impacted by some major event which destroyed habitat and reduced populations to small sizes. There is only a single haplotype found on Sagalla despite wide sampling throughout the area (although sample sizes were low), and the lack of haplotype diversity suggests a very recent demographic event, rather than a historical one. Indigenous forest on Sagalla has been reduced to around 4 ha due to anthropogenic activities, a much higher reduction than any of the other montane isolates [58]. Indeed, other forest species are thought to have gone extinct following the replacement of the indigenous forest with pine [49], and habitat destruction associated with these activities could possibly have reduced the Sagalla population to low numbers with little diversity.

We have assumed that the single haplotype from Mbololo found in several places in the eastern area of Dawida (Fig. 3A) is the result of recent anthropogenically aided translocation. People from the Taita Hills are unlikely to deliberately move chameleons as they are not held in high esteem in local belief systems [59], but because *K. boehmei* lay eggs in soil, it is quite likely that these have been moved between montane isolates together with the rootstock of plants. Had this been the result of natural colonization or shared ancestral polymorphism, we would have shared haplotypes between both populations (no Dawida haplotypes were found on Mbololo), and/or fewer mutational steps between the shared haplotype. Most of the individuals on Dawida with the “Mbololo haplotype” were found in a plantation closest to Mbololo, rather than natural forest. In addition, all chameleons were found on the eastern side of Dawida which is closest to the town of Wundanyi, the main trading centre on Dawida.

### Conclusions

Here we present an historical scenario of Pleistocene vicariance in a forest chameleon now restricted to isolated fragments on inselbergs of the northernmost Eastern Arc Mountains. The most recent vicariant events coincide with end of the last particularly wet episode of three wet periods during continuing post-Miocene aridification in East Africa [48]. Tolley et al [43] suggested that vicariance due to forest fragmentation starting in the Oligocene has driven speciation patterns of the genus *Kinyongia*. Our data concur that without forest connections, forest adapted chameleons are not capable of moving even small distances across the dry savannah. In addition, we provide insight into the dispersal of these arboreal lizards during the Pleistocene. As these chameleons are forest dependent, we presume that gene exchange during this episode was made possible by continuous forest between montane isolates. Thus while other East African taxa saw radiations during this period [53], [54], these chameleons managed only to disperse relatively short distances. Lastly, a population expansion event on Dawida was dated to approximately 182 ka which we suggest represents re-colonisation from a remnant population.

## Materials and Methods

### Ethics statement

Permission to collect and sample *Kinyongia boehmei* in the Taita Hills was obtained from the Ministry of Education Science and Technology (research permit number MOEST 13/001/36C 183), the National Museums of Kenya, Kenya Wildlife Service, the Taita-Taveta district officer and the Kenyan Ministry of Forestry Taita-Taveta division.

### Study species

The Taita two-horned chameleon, *Kinyongia boehmei*, was previously known from two of the four Taita montane isolates [42], but in this study we confirm its presence on all four Taita Hills above 950 m asl. Males have striking bladed horns which they use in combat to wrestle each other from braches, deposed males being thrown to the forest floor. Females have reduced horns, are most often green with a reddish-brown casque, and lay clutches of six to 12 eggs in nests beneath the forest floor [59]. Although their primary habitat is Afromontane forest, these chameleons can be found in afforested areas outside of indigenous forest fragments [60], including plantations (*Eucalyptus, Cypressa* and *Pinus* spp.) and small-scale agroforestry on small holdings (*Eucalyptus* spp. and *Gravillia robusta*). Nothing is known of population level gene-flow for any species in this genus. This species is known to be most closely related to *Kinyongia tavetana* which is distributed in North Pare (part of the EAM) as well as the volcanoes Kilimanjaro and Meru [43]. *K. tavetana* is also known from the Chuylu Hills [59], although the genetic relationship of this population is not known.

### Sampling

Tissue samples of chameleons (2 mm tail tips preserved in 98% ethanol; Herrel et al in press) were obtained during field surveys in the Taita Hills from 2002 to 2008. Samples were concentrated around indigenous forest fragments, as well as formerly forested transformed land (plantations and small holdings) on the montane isolates of Dawida, Mbololo, Sagalla and Kasigau in the Taita Hills, Kenya (Fig. 1, 3A). Chameleon samples were taken simultaneously with locality data using a Garmin 12XL GPS. Euclidian distances between montane isolates were calculated in ARCVIEW 9.0 (ESRI). We obtained tissue samples from 146 individuals from all four montane isolates of the Taita Hills, representing the entire known range [42], [59], and an extension of the known range into the more distant Sagalla and Kasigau montane isolates.

### DNA isolation and sequencing

Total genomic DNA was isolated using a standard salt extraction [61]. To construct a phylogenetic tree of Taita Hills *Kinyongia* we sequenced two mitochondrial (16 S and ND2) and two nuclear markers (RAG1 and phosducin) for three individuals from each montane isolate and two outgroup individuals (*Bradypodion pumilum* and *Kinyongia tavetana*). Next, we sequenced all remaining individuals following Tolley et al. [46] in their study of South African dwarf chameleons (*Bradypodion* spp.) by choosing the ND2 marker which was found to have variation at the population level. This gave us a second data set of 146 individuals for the population level analysis. Lastly, for estimates of divergence time, we utilised sequences from the three genes with reasonable estimates of mutation rates (ND2, 16 S, RAG). To minimise effects of unequal sample sizes, and to avoid intractable calculations for divergence time estimates, 19 randomly selected individuals were included from Dawida with all individuals included from Mbololo (n = 17), Sagalla (n = 15) and Kasigau (n = 4).

PCR conditions were as follows: ND2 using primers L4349 (designed for this study: 5′ GGG GCT ACT TTG ATA GAG 3′) and H5934 [62]. PCR annealing temperature 54°C (40 cycles), 1.5 mM MgCl_2_; 16 S using primers 16 Sa and 16 Sb [63] annealing at 52°C (35 cycles) 0.75 mM MgCl_2_; phosducin Phos R1 and Phos F2 [64] annealing at 52°C (35 cycles), 1.5 mM MgCl_2_; RAG1 using primers F118 and R1067 [65] annealing at 57 C (40 cycles), 1.5 mM MgCl_2_. Amplified PCR products were sent to Macrogen Inc., Korea for sequencing. Sequences were checked and aligned with GeneiousPro v 4.8 [66]. All new sequences have been deposited in EMBL Nucleotide Sequence Database (HE601966–HE602017).

### Population genetic analyses

A spatial analysis of variance (SAMOVA v1.0) was conducted on the more comprehensive ND2 data set to examine clusters of sampling sites that are maximally differentiated but geographically homogenous [67]. This analysis uses haplotype data and geographic co-ordinates of each individual chameleon sampled to statistically differentiate between clusters of sample sites that presumably represent populations. The SAMOVA was run for K = 2 to 8 groups to determine the maximum value of F_CT_, the maximized proportion of total genetic variance due to differences between clusters [67]. Subsequently, haplotype (*h*) and nucleotide (π) diversity were estimated in ARLEQUIN (v3.5 [68]) for each population determined by SAMOVA.

Relationships among haplotypes were examined using ND2 haplotype data with a median-joining network in Network 4.1 [69] and a split NeighborNet algorithm [70] in SplitsTree v4.11.3 [71]. The median-joining network revealed a pattern of possible secondary contact between Dawida and Mbololo, as highly divergent haplotypes are shared across the mountains (Fig. 3). As this result had implications for our dating methods as well as hypotheses relating to dispersal, we subsequently conducted population analysis both with and without these instances of possible secondary contact.

To examine whether populations on each mountain have experienced historical demographic changes, Fu's *F_S_* was used to estimate whether populations were out of mutation–drift equilibrium [72], [73] using Arlequin 3.5 [68]. In the case of a recent demographic shift, such as a population expansion, a significant negative value would be obtained (Fu 1997,[74]. For any populations out of equilibrium, we then estimated the timing of the demographic shift by applying a model of demographic expansion [68], [75], [76]; *t* = τ/(2*u*), where *t* is time in generations, τ is the age of the expansion in mutational units (estimated in the model of demographic expansion) and u is the sum of the per nucleotide mutation rate for the region sequenced [75], [76]. For the present study, generation time was estimated at 2 years (based on information from captive breeding) and *u* was obtained from the same estimate of mutation rate as described for coalescence analysis (below).

The pattern of forest fragmentation was examined by testing for isolation by distance (IBD) using the Mantel test. We hypothesised that sequential forest fragmentation between mountains would display an IBD pattern; i.e. proximal forests would stay connected longer than distant ones (see Fig. 1). We used pairwise *F_ST_* values from the ND2 dataset together with Euclidian distances between montane isolates in a Mantel test with 10 000 permutations in ARLEQUIN (v3.5 [68]). Alternatively, if mountains remained connected (e.g. through riverine corridors), divergence times (below) for those pairs of mountains would be more recent than for those pairs which had fragmented earlier and not remain connected.

### Divergence times

To estimate divergence times between montane isolates, a coalescent approach was used, incorporating the Isolation with Migration (IM) model using the software IMa2 (v 2.0 [77]). This analysis requires an input topology for the sampled populations as a prior, and this was obtained through a Bayesian and maximum likelihood analysis (see below). The IMa2 software uses a Felsenstein framework to run Markov chain Monte Carlo (MCMC) simulations permitting likelihood-based analyses [78]. MCMC parameters of the model included a burn-in duration of 2.5 million steps, 25 000 genealogies saved and a geometric heating model with 20 chains, 0.95 as first and 0.9 as second chain heating parameters. Priors were set by running analysis trials (maximum population size = 200, maximum migration = 0, maximum time of population splitting = 8). Three final duplicate runs were submitted to the remote computer cluster running the program IMa2 at Cornell University via internet upload (http:cbsuapps.tc.cornell.eduIMa.aspx). The model parameter *t* was obtained for each comparison, as were marginal posterior probability densities with 95% upper and lower limits [77]. To estimate divergence time in years (T), the geometric mean of the mutation rate (U) for the markers sequenced were used: T = *t*/U [78]. Mutation rates for each marker (μ) per site per million years were first estimated by applying the divergence time of 14 Myr between *K. boehmei* and the sister species *K. tavetana* [43] to the accompanying gene specific sequence divergences (Appendix S3 from [43]). To provide estimates of uncertainty in the dates, a range of mutation rates were incorporated, based on means and 95% highest probability densities of divergence times (Appendix S1 from [43]).

### Tree topology construction for coalescent prior

Analysis using Isolation with Migration (IM) model using the software IMa2 (v 2.0 [77]) requires an input topology as a prior. In order to obtain this topology, we conducted a Bayesian analysis of 2 270 characters from two mitochondrial markers (ND2, 783 bp and 16 S, 451 bp) and two nuclear markers (RAG1, 700 bp; phosducin 333 bp) to investigate optimal input tree space using MrBayes 3.1.0 [79]. Several data partitions were created, which were unlinked and allowed to run with separate values for the model parameters. A single data partition was created for 16 S, three partitions for each of the coding genes (ND2, RAG1, phosducin) 1st, 2nd, and 3rd codons separately. To examine whether the model used was over-parameterised, an additional MCMC was run with only 4 partitions (one for each marker). For each marker, Modeltest was initially run to investigate the evolutionary model that best fits the data. Both the AIC and LRT tests were used to guide the choice of rate categories for the partitions, and where the tests differed, the more simple model was chosen (16 S, nst = 2+G; ND2, nst = 2+G; RAG1, nst = 2+I+G; phosducin, nst = 1+G). To ensure the results converged on the same topology, the MCMC was run in parallel, twice for each of the two model variations for 10 million generations each. Trees were sampled every 1000 generations and the first 1 million generations (1000 trees) were removed as burn-in, after examining the average standard deviation of split frequencies (<0.001), the convergence diagnostic (PSRF values ∼1.0) as well as the log-probabilities and the values of each parameter for stabilisation [80]. For each run, the effective sample size (ESS) for all parameters was checked using Tracer v. 1.4.1 to ensure that ESS >200 [81]. A 50% majority rule tree was constructed and nodes with >0.95 posterior probability considered as supported. In addition, a partitioned maximum likelihood (ML) search was run in GARLI-PART 0.97 [82], using the same models and partitions as above. All parameters were estimated, and a random starting tree was used. One hundred bootstrap replicates were run to evaluate confidence in the nodes. Nodes with a bootstrap value of ≥70% were considered supported in this analysis. These analyses were run three times to ensure that independent ML searches produced the same topologies. As an additional metric for comparison with other species of chameleons, uncorrected p-distances for each marker were estimated between each montane isolate.
